# Novel chemotherapeutic agent FX-9 activates NF-κB signaling and induces G1 phase arrest by activating *CDKN1A* in a human prostate cancer cell line

**DOI:** 10.1186/s12885-021-08836-y

**Published:** 2021-10-08

**Authors:** F. Weiner, J. T. Schille, D. Koczan, X.-F. Wu, M. Beller, C. Junghanss, M. Hewicker-Trautwein, H. Murua Escobar, I. Nolte

**Affiliations:** 1 Small Animal Clinic, University of Veterinary Medicine Hannover, 30559 Hannover, Germany; 2 Department of Medicine, Clinic III, Hematology, Oncology, Palliative Medicine, University of Rostock, 18057 Rostock, Germany; 3 Core Facility for Microarray Analysis, Institute for Immunology, University of Rostock, 18057 Rostock, Germany; 4 Leibniz Institute for Catalysis, 18059 Rostock, Germany; 5 Department of Pathology, University of Veterinary Medicine Hannover, 30559 Hannover, Germany; 6 Comprehensive Cancer Center – Mecklenburg Vorpommern (CCC-MV), Campus Rostock, University of Rostock, 18057 Rostock, Germany

**Keywords:** Isoquinolinamine FX-9, Antimitotic agent, Microarray analysis, NF-κB signaling, G1-phase arrest, Prostate cancer

## Abstract

**Background:**

The aminoisoquinoline FX-9 shows pro-apoptotic and antimitotic effects against lymphoblastic leukemia cells and prostate adenocarcinoma cells. In contrast, decreased cytotoxic effects against non-neoplastic blood cells, chondrocytes, and fibroblasts were observed. However, the actual FX-9 molecular mode of action is currently not fully understood.

**Methods:**

In this study, microarray gene expression analysis comparing FX-9 exposed and unexposed prostate cancer cells (PC-3 representing castration-resistant prostate cancer), followed by pathway analysis and gene annotation to functional processes were performed. Immunocytochemistry staining was performed with selected targets.

**Results:**

Expression analysis revealed 0.83% of 21,448 differential expressed genes (DEGs) after 6-h exposure of FX-9 and 0.68% DEGs after 12-h exposure thereof. Functional annotation showed that FX-9 primarily caused an activation of inflammatory response by non-canonical nuclear factor-kappa B (NF-κB) signaling. The 6-h samples showed activation of the cell cycle inhibitor *CDKN1A* which might be involved in the secondary response in 12-h samples. This secondary response predominantly consisted of cell cycle-related changes, with further activation of *CDKN1A* and inhibition of the transcription factor *E2F1*, including downstream target genes, resulting in G1-phase arrest. Matching our previous observations on cellular level senescence signaling pathways were also found enriched. To verify these results immunocytochemical staining of p21 Waf1/Cip1 (*CDKN1A*), E2F1 (*E2F1*), PAI-1 (*SERPNE1*), and NFkB2/NFkB p 100 (*NFKB2*) was performed. Increased expression of p21 Waf1/Cip1 and NFkB2/NFkB p 100 after 24-h exposure to FX-9 was shown. E2F1 and PAI-1 showed no increased expression.

**Conclusions:**

FX-9 induced G1-phase arrest of PC-3 cells through activation of the cell cycle inhibitor *CDKN1A*, which was initiated by an inflammatory response of noncanonical NF-κB signaling.

**Supplementary Information:**

The online version contains supplementary material available at 10.1186/s12885-021-08836-y.

## Background

The amino-substituted isoquinoline FX-9 (3-(p-Tolyl)isoquinolin-1-amine) [1] belongs to a substance family showing antimalarial [2], and antifungal [3] properties as well as activity against different tumors [4–6]. Previous studies by us proved anti-proliferative effects of FX-9 on hematological and solid tumor cell lines. This effect was accompanied with morphological changes and apoptosis in lymphoblastic leukemia cells, while cytotoxicity and hemolytic activity against non-neoplastic blood cells were not observed [7]. Furthermore, the effects on prostate cancer cells lines of human and canine origin were pro-apoptotic and antimitotic. This was evaluated by analyzing cell viability, total cell number, cell morphological changes and induction of apoptosis [8]. FX-9-mediated cytotoxic activity in non-malignant chondrocytes and fibroblasts was found to be decreased [8].

Detailed knowledge of the molecular mechanism of new potential chemotherapeutic agents is paramount in order to predict safety, efficacy, possible adverse effects, and resistance [9]. Key tools to reveal the molecular mode of action are microarrays and RNA-sequencing, allowing identification of differentially expressed genes (DEGs).

Antimitotic drugs disrupt the cell cycle progression of tumor cells. The purpose of the cell cycle is to duplicate the DNA accurately and to segregate the copies into two identical daughter cells. In brief, the cell cycle is divided into G1-phase, S-phase, G2-phase and M-phase. It is a complex process whose successful progress is controlled by many checkpoints in healthy cells. If a checkpoint is activated, for example, due to DNA damage, the cell cycle is arrested until either DNA repair or programmed cell death occurs [10]. In tumor cells, the checkpoints are often defective, making them selective for drugs that target these checkpoints [11]. Possible targets are cyclin-dependent kinases (CDKs), the regulators of cell cycle progression [12]. CDKs form complexes with cyclins [13] and can be inhibited by the cyclin-dependent kinase inhibitor 1A (CDKN1A, p21). Since its discovery in 1993 [14], research on involvement of CDKN1A in cell cycle, cell differentiation, and apoptosis is of great interest [15, 16].

PC-3 is a human prostate cancer cell line, representing the castration-resistant prostate cancer. It develops in 10–20% of patients with prostate cancer where androgen deprivation therapy has failed [17]. The cell line PC-3 is a stable and well-characterized androgen-independent human cell line. The *in vivo* behavior of this cell line is also well known, so adapting research on FX-9 to in vivo experiments would be simplified.

The aim of the present study was to characterize in vitro the molecular mode of action of FX-9 by gene expression level in a stable in vitro model system (PC-3 cell line) and verification of selected targets at protein level.

## Methods

### FX-9

FX-9 (3-(p-Tolyl)isoquinolin-1-amine) was synthesized by Feng and Wu [1]. The 10 mM stock solution was dissolved in dimethylsulfoxide (DMSO; Merck KGaA, Darmstadt, Germany) and stored at − 20 °C. For the experiments a final concentration of 5 μM was used in accordance with our previous results [8] and prepared immediately prior to each experiment.

### Cell lines and cell culture

PC-3 is an androgen-insensitive human cell line of a bone metastasis from a prostate carcinoma of a 62-year-old man [18]. The cell line was cultivated in 25 cm^2^ cell culture flasks in medium 199 (Gibco™, Thermo Fisher Scientific, Inc., Waltham, MA, USA) with 10% fetal bovine serum superior (Biochrom GmbH, Berlin, Germany) and 2% penicillin-streptomycin (Biochrom GmbH). The cells were cultivated at 37 °C and 5% CO_2_ in a humidified atmosphere.

### RNA-isolation and –integrity

The cell lines were seeded at a density of 600,000 cells in 5 ml culture medium in 25 cm^2^ cell culture flasks and incubated for 12 h at 37 °C and 5% CO_2_ in a humidified atmosphere. The cells were exposed to 5 μM FX-9 for 6 and 12 h, cultured in an incubator at 37 °C and 5% CO_2_ in a humidified atmosphere. The concentration was selected as it had shown significant anti-proliferative effects on PC-3 in our previous study [8]. Time points were chosen in accordance with our previous cell biological results characterizing early transcriptome alteration before induction of cell death. Untreated cells served as control. The cells were detached with a cell scraper and collected in phosphate-buffered saline. The cell suspension was transferred into tubes and centrifuged at 20 °C and 150 x g for 10 min. The supernatant was discarded, the cell pellets were lyzed using 600 μl chaotropic buffer (RLT plus buffer, Qiagen GmbH, Hilden, Germany). The lyzates were transferred into cryotubes and stored at − 80 °C. RNA extraction was performed using the RNeasy Plus Kit (Qiagen GmbH) including a DNA removal step in accordance with the manufacturer’s protocol. The RNA samples were quantified spectrophotometrically (Nanodrop 1000 (Thermo Fisher Scientific, Inc., Waltham, MA, USA)) and diluted to a concentration of 70 ng per microliter for RNA integrity analysis (Agilent RNA 6000 Nano Chip using Bioanalyzer 2100 instrument (Agilent Technologies, Inc., Santa Clara, Ca, USA)). Samples were prepared as independent biological replicates. All samples showed an RNA integrity number of 10.0.

### Gene expression level profiling

200 ng RNA was used as starting material in the GeneChip^R^ Whole Transcript Sense Target Labeling protocol (Affymetrix, Thermo Fisher Scientific, Inc.). The microarray hybridization was performed using the Affymetrix Clariom™ S Array Kit in accordance with the manufacturer’s instructions (Affymetrix, Thermo Fisher Scientific, Inc.): in detail, the so called WT (Whole Transcriptome) protocol started with first strand synthesis by introducing T7 promoter tags to all RNA molecules using N6 3` ends. After strand replacement in accordance with Eberwine, non-labeled aRNA (antisense RNA) was produced by in vitro transcription in agreement with the linear amplification of all RNA molecules without a 3’bias. After an aRNA cleanup (magnet bead based), a new strand identical single-strand DNA was produced using the aRNA as template by adding random primers and deoxyribonucleoside 5′-triphosphates (dNTPs). In the meantime, a certain amount of dTTP was replaced by dUTP. After removing the aRNA by RNaseH digestion and clean-up (magnet bead based), this enabled an enzymatic endpoint fragmentation. Therefore, uracyldeglycosidase removed the uracils in combination with APE1 (apurinic apyrimidinic endonuclease 1), which is cleaved the deuracylized phosphodiester backbone of the single strand DNA molecules. Desoxynucleotidyltransferase was added to the DNA labeling reagent (Biotin-11-dXTP) to the 3` ends of the single strand DNA fragments. The hybridization was carried out overnight (16 h) at 45 °C in the GeneChip^R^ Hybridization Oven 645 (Affymetrix, Thermo Fisher Scientific, Inc.). Washing and staining protocols, including an antibody amplification, were applied by the GeneCip Fluidis Station 450. The microarrays were scanned using the GeneChip Scanner 3000 7G (Affymetrix, Thermo Fisher Scientific, Inc.) at 0.7 μm resolution.

### Analysis of the microarray data

Affymetrix Clariom™ S Arrays interrogate more than 20,800 genes using about 800,000 probes. The data was analyzed by the Transcriptome Analysis Console (TAC) version 4.0.2.15 (Applied Biosystems, Thermo Fisher Scientific, Inc.) with the implemented SST-RMA normalization algorithm. Replicate groups were summarized by LIMMA statistics. Differentially expressed genes (DEGs) were identified using filter parameters fold change (FC) < 2 or < − 2, LIMMA *p*-value < 0.05, and FDR-value < 0.05.

### Analysis of signaling pathways and biological processes

To analyze the cellular signaling pathways effected by FX-9, the total number of DEGs were examined in the known pathway network Reactome [19]. Analyzing DEGs by gene ontology functional processes was performed via the gene network Database for Annotation, Visualization and Integrated Discovery (DAVID, DAVID Bioinformatics Resources 6.8) [20]. DEGs enriched signaling pathways with an FDR-value < 0.05 were considered for further analysis.

### Immunocytochemical (ICC) staining

PC-3 cells were seeded at a density of 5 × 10^6^ cells per 75 cm^2^ culture flask and were incubated over night to adhere. After exposure to 5 μM FX-9 or cell culture medium for control for 12 h or 24 h, the cells were harvested by TrypLE™ Express Enzyme (Gibco™, Thermo Fisher Scientific, Inc.) and pelleted in 1.5 ml tubes by centrifugation (20 °C, 150 x g, 10 min). The cell pellets were fixed in 1 ml cold 4% paraformaldehyde and stored at 4–8 °C for a maximum of 7 days until embedding in paraffin. After slicing the paraffin-embedded cell pellets, they were mounted on slides. Thermal antigen demasking in citrate buffer was performed for 20 min in a microwave. To exclude non-specific binding, the slides were blocked with 20% goat serum for 20 min. This was followed by incubation with mouse monoclonal primary antibodies p21 Waf1/Cip1 (diluted 1:100, sc-6246, Santa Cruz Biotechnology, Inc., Dallas, TX, USA,), E2F-1 (diluted 1:200, sc-251, Santa Cruz Biotechnology, Inc.,), PAI-1 (diluted 1:50, sc-5297, Santa Cruz Biotechnologys, Inc.), and rabbit monoclonal primary antibody NFkB2/NFkB p100 (diluted 1:200, JM82–03, Novus Biologicals, LLC, Littleton, CO, USA) at 4 °C over night. After a 45-min incubation period with biotinylated secondary antibodies goat anti-mouse (diluted 1:200, BA-9200, Vector Laboratories, Inc., Burlingame, CA, USA) or goat anti-rabbit (diluted 1:200, BA-1000, Vector Laboratories, Inc.,) indirect avidin-biotin-peroxidase staining with VECTASTAIN®Elite® ABC-kit (Vector Laboratories, Inc.) was performed in accordance with manufacturer’s protocol. Reaction was visualized by incubation in 0.1 g 3,3′-diaminobenzidine tetrahydrochloride (DAB, Acros Organics, BVBA, Fair Lawn, NJ, USA) in 200 ml PBS and counterstaining was performed with hematoxylin. The experiment was performed in triplicates. Staining intensities and the fraction of positively stained cells were scored as proposed by Sorenmo et al. [21] and Pagliarone et al. [22] by an experienced and blinded pathologist.

## Results

### FX-9 caused changes in gene expression level profile at 6-h and 12-h exposure

**Fig. 1:**
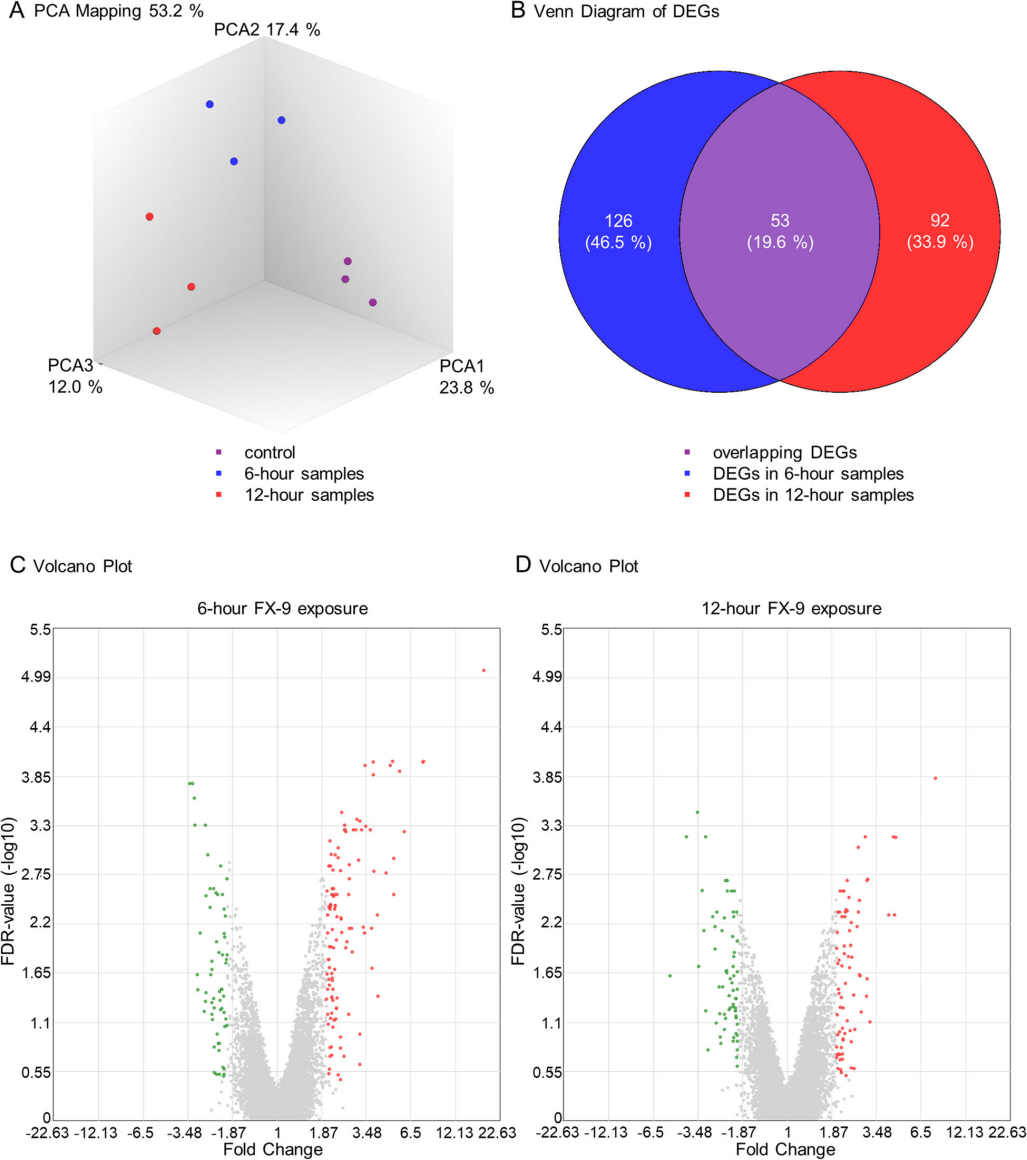
**A**: PCA mapping: Three-dimensional diagram: clear differentiation of the three sample groups showing high consistency within the individual replicates in the original dataset. **B**: Venn diagram: Number of DEGs in 6-h and 12-h samples are displayed and overlapping DEGs (*n* = 53) are highlighted in purple. **C and D**: Volcano plots after 6-h and 12-h exposure of 5 μM FX-9. Plots display the FC against the negative decadic logarithm of the FDR-value. Color code: red FC > 2, green FC < − 2, both with LIMMA *p*-value < 0.05, and FDR-value < 0.05

Principal component analysis (PCA) with TAC software showed clustering of the three different sample groups: control, 6-h and 12-h samples (Fig. 1). A total of 21,448 genes were analyzed by the microarray. PC-3 expressed 18,722 of these genes, and after 6-h exposure to 5 μM FX-9, 179 genes were differentially expressed compared to untreated controls. In comparison to the total number of genes analyzed, this represented 0.83%. In relation to the genes expressed in PC-3, the percentage of DEGs was 5.24%. Of these DEGs, 116 genes showed increased expression with fold change ranging from 2 to 18.05. The expression of 78 genes was decreased with fold change between − 2 and − 3.41.Gene expression analysis after exposure to 5 μM FX-9 over a 12-h period revealed a total of 145 DEGs compared to non-treated control samples. This represented that 0.68% of the total analyzed genes and 0.75% of the 19,280 expressed genes in PC-3 in the 12-h samples. The expression of 78 genes was increased between fold change of 2 and 7.92 and the expression of 67 genes was decreased within fold change ranging from − 2 to − 5.14. On comparing DEGs of 6-h and 12-h samples, overlapping of 53 DEGs was shown. The annotation of the DEGs of the 6-h and 12-h samples to signaling pathways is described in the following paragraphs of the results.

### FX-9 induced Reactome-pathways of the immune system after 6-h exposure and in cell cycle pathways after 12-h exposure

**Fig. 2:**
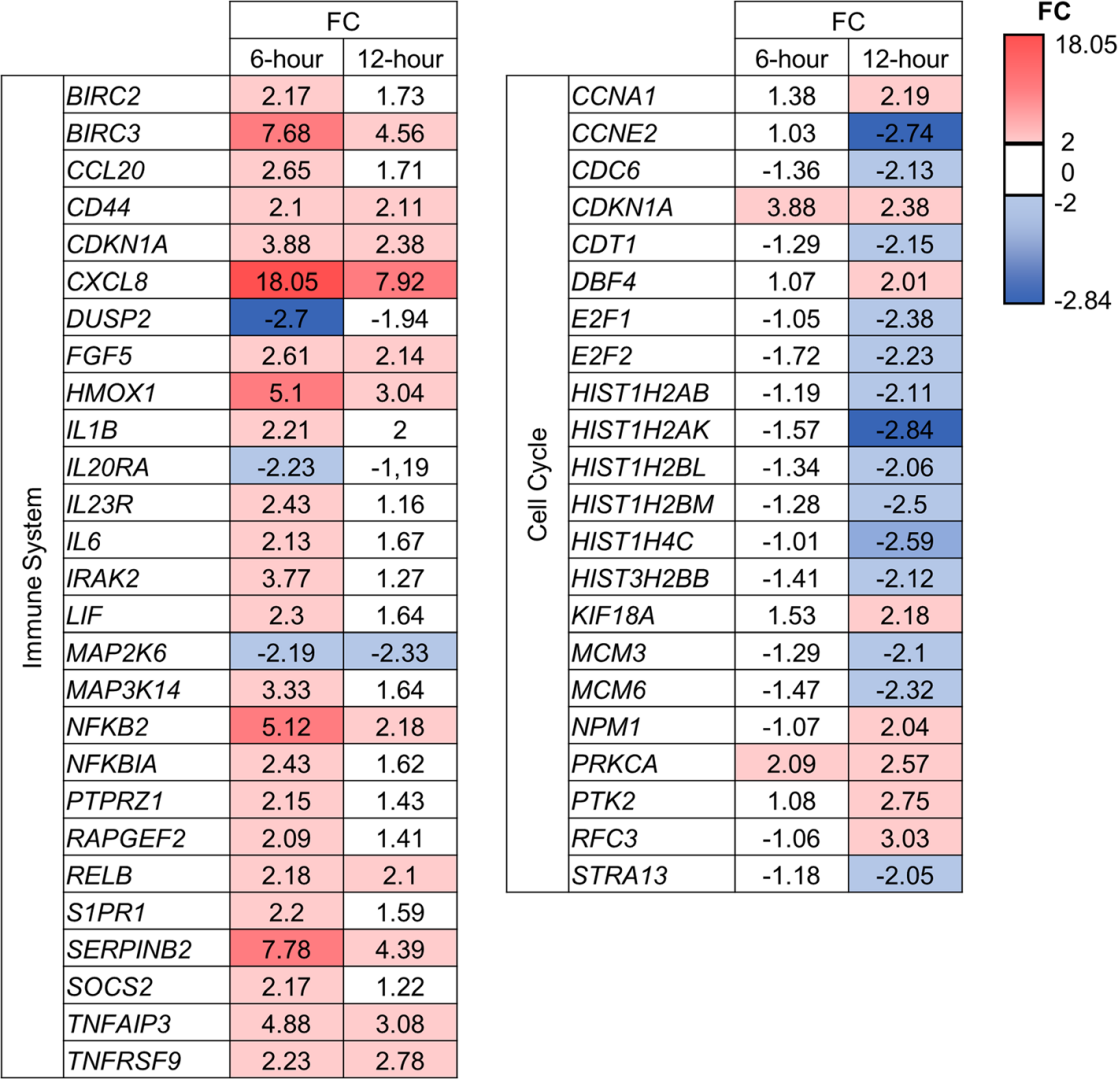
DEGs belonging to Reactome pathways “Immune System”, and “Cell Cycle” after 6-h and 12-h FX-9 exposure. Numbers given for each gene display the FC (in linear space) of 6-h and 12-h samples versus the control. Values highlighted in red or blue indicate significantly higher or lower expression. Non-highlighted values are not significantly different (LIMMA *p*-value < 0.05, and FDR-value < 0.05)

After identifying the DEGs, signaling pathway analysis was performed using the gene database Reactome. The total list of DEGs was submitted to Reactome, where the DEGs were mapped to signaling pathways and analyzed for over-represented signaling pathways. The analysis was performed statistically using a hypergeometric distribution that identified enriched signaling pathways in the submitted data. Fifteen signaling pathways passed the criteria of FDR-value < 0.05. Eight of these fifteen enriched signaling pathways can be assigned to the superpathway “immune system” (Table 1). These signaling pathways were mainly activated by increasingly expressed genes. Two signaling pathways of the superpathway “cell cycle” were enriched by upregulation of *CDKN1A* (FC 3.88, Fig. 2). The pathway “TP53 regulates transcription of genes involved in G1 cell cycle arrest” belongs to the superpathway gene expression, describes the involvement of genes in G1-phase cell cycle arrest and was enriched by activation of *E2F7* (FC 2.56). Two signaling pathways were activated by increasingly expressed genes and functionally belong to the superpathway “signal transduction”. The signaling pathway “insulin-like growth factor-2 mRNA binding proteins (IGF2BPs/IMPs/VICKZs) bind RNA” belongs to the superpathway “RNA metabolism”, and was enriched by two DEGs. The signaling pathway “cellular senescence”, which belongs to the superpathway “cellular response to external stimuli” was activated by seven DEGs.

**Table 1:** Reactome top 15 enriched signaling pathways after 6-h exposure of FX-9

No.	Super-pathway	Signaling Pathway	DEGs	Entities	
				found	FDR
1	IS	Interleukin-4 and Interleukin-13 signaling	8	17/216	2.24e-05
2	MR	Insulin-like Growth Factor-2 mRNA Binding Proteins (IGF2BPs/IMPs/VICKZs) bind RNA	2	6/13	2.24e-05
3	IS	Interleukin-10 signaling	5	11/86	2.73e-05
4	IS	Signaling by Interleukins	17	29/647	4.07e-05
5	IS	Cytokine Signaling in Immune system	26	37/1108	7.63e-04
6	GE	TP53 Regulates Transcription of Genes Involved in G1 Cell Cycle Arrest	2	5/20	0.002
7	ST	Antagonism of Activin by Follistatin	3	3/4	0.003
8	ST	Signaling by TGFB family members	7	9/114	0.006
9	CC	Transcriptional activation of cell cycle inhibitor p21	1	3/6	0.008
10	CC	Transcriptional activation of p53 responsive genes	1	3/6	0.008
11	IS	CLEC7A/inflammasome pathway	1	3/8	0.015
12	IS	Interleukin-1 processing	2	3/8	0.015
13	CR	Cellular Senescence	7	11/200	0.015
14	IS	NOD1/2 Signaling Pathway	5	5/40	0.02
15	IS	Nucleotide-binding domain, leucine rich repeat containing receptor (NLR) signaling pathways	6	6/70	0.035

Superpathways: *IS* “immune system”, *MR* “metabolism of RNA”, *GE* “gene expression (RNA polymerase II transcription)”, *CC* “cell cycle”, *ST* “signal transduction”, and *CR* “cellular response to external stimuli”. The found entities display the number of entries of the DEGs in the signaling pathway.

Analysis of DEGs comparing control with 12-h exposure of 5 μM FX-9 showed 119 affected signaling pathways with an FDR-value < 0.05. Of these, the top fifteen enriched signaling pathways were considered for further analysis (Table 2). Seven signaling pathways assigned to the superpathway “cell cycle” were enriched by DEGs. These signaling pathways were inhibited by genes involved in the transition of cells from the G1- to the S-phase, such as *CCNE2*, *CDC6*, *CDKN1A*, *CDT1*, *E2F1*, *E2F2*, *MCM3* and *MCM6* (Fig. 2). The two pathways belonging to the superpathway “gene expression” are also involved in the cell cycle. These were enriched by DEGs *CCNE2*, *CDKN1A*, *E2F1*, *E2F2*, and *E2F7*. DEGs enriched three signaling pathways belonging to the superpathway “cellular response to external stimuli”. The signaling pathways “DNA replication” and “transcriptional regulation of granulopoiesis” were enriched by DEGs similar to those in the superpathway “cell cycle”. In the superpathway “RNA metabolism”, the signaling pathway “Insulin-like Growth Factor-2 mRNA Binding Proteins (IGF2BPs/IMPs/VICKZs) bind RNA” was enriched by upregulation of *CD44.*

**Table 2:** Reactome top 15 enriched signaling pathways after 12-h exposure of FX-9

No.	Super-pathway	Signaling Pathway	DEGs	Entities	
				found	FDR
1	GE	TP53 Regulates Transcription of Genes Involved in G1 Cell Cycle Arrest	6	9/20	8.77e-09
2	CR	Cellular Senescence	14	17/200	8.10e-07
3	CR	DNA Damage/Telomere Stress Induced Senescence	10	11/71	1.06e-06
4	CC	Mitotic G1 phase and G1/S transition	10	15/173	2.85e-06
5	GE	TP53 Regulates Transcription of Cell Cycle Genes	7	10/65	3.46e-06
6	CC	Cell Cycle	22	30/734	5.34e-06
7	CC	Cell Cycle Checkpoints	14	18/280	5.34e-06
8	CC	Cell Cycle, Mitotic	19	25/596	3.98e-05
9	CR	Senescence-Associated Secretory Phenotype (SASP)	9	10/91	4.00e-05
10	R	DNA Replication	10	12/142	4.00e-05
11	DB	Transcriptional regulation of granulopoiesis	10	9/71	4.00e-05
12	CC	Deposition of new CENPA-containing nucleosomes at the centromere	8	8/54	4.56e-05
13	CC	Nucleosome assembly	8	8/54	4.56e-05
14	CC	G1/S Transition	10	12/150	5.11e-05
15	MR	Insulin-like Growth Factor-2 mRNA Binding Proteins (IGF2BPs/IMPs/VICKZs) bind RNA	1	5/13	5.11e-05

Superpathways: *GE* “gene expression (RNA polymerase II transcription)”, *CR* “cellular response to external stimuli”, *CC* “cell cycle”, *R* “DNA replication”, *DB* “developmental biology”, and *MR* “metabolism of RNA”. The found entities display the number of entries of the DEGs in the signaling pathway.

### FX-9 induced time-dependent differences in the biological processes of inflammatory response and G1/S transition of mitotic cell cycle

To assign DEGs to the context of the affected biological processes in the cells, the platform DAVID was used. Biological processes with an FDR-value < 0.05 were included in this analysis. The processes “inflammatory response”, “angiogenesis, positive regulation of cell migration”, “positive regulation of angiogenesis”, “extracellular matrix organization”, and “negative regulation of apoptotic process” were enriched by DEGs at both time points (Table 3). The process with the lowest FDR-value of 0.00013 in the 6-h samples was the “inflammatory response” affected by 18 DEGs. This process was also enriched in the 12-h samples, but with a higher FDR-value of 0.027 and 12 DEGs.

**Table 3:** DAVID analysis functional annotation, gene ontology, 6-h and 12-h FX-9 exposure

GO.biological processes	6-h		12-h	
	DEGs	FDR	DEGs	FDR
inflammatory response	18	1.30E-04	12	2.70E-02
angiogenesis	14	1.30E-04	13	1.70E-04
positive regulation of cell migration	12	4.80E-04	11	6.00E-04
positive regulation of angiogenesis	10	4.80E-04	9	8.40E-04
I-kappaB kinase/NF-kappaB signaling	7	5.30E-03		
negative regulation of cell proliferation	15	5.30E-03		
positive regulation of NF-kappaB transcription factor activity	9	7.40E-03		
positive regulation of transcription from RNA polymerase II promoter	24	8.00E-03		
extracellular matrix organization	10	1.40E-02	10	4.80E-03
negative regulation of apoptotic process	15	1.40E-02	14	9.40E-03
cell adhesion	15	1.40E-02		
positive regulation of nitric-oxide synthase biosynthetic process	4	2.50E-02		
positive regulation of ERK1 and ERK2 cascade	9	2.70E-02		
G1/S transition of mitotic cell cycle			9	6.00E-04
positive regulation of endothelial cell proliferation			6	2.70E-02

After 6-h exposure of FX-9, genes with increased expression enriched the processes “I-kappaB kinase/NF-kappaB signaling”, “positive regulation of NF-kappaB transcription factor activity”, “negative regulation of cell proliferation”, and “cell adhesion”. In the biological process “positive regulation of transcription from RNA polymerase II promoter”, of a total of 24 DEGs, 18 showed positive and 6 negative FC. The biological process “positive regulation of nitric-oxide synthase biosynthetic process” was also enriched by nine DEGs. The biological process “positive regulation of ERK1 and ERK2 cascade” was enriched by upregulated genes with two exceptions. The biological processes “G1/S transition of mitotic cell cycle” and “positive regulation of endothelial cell proliferation” were only enriched in the 12-h samples after FX-9 exposure. “G1/S transition of mitotic cell cycle” was enriched with an FDR-value of 0.0006 by nine genes, including *CDC6*, *CDT1*, *CDKN1A*, *CCNE2*, *MCM3*, and *MCM6*.

### FX-9 application increased the amount of CDKN1A (p21) protein expressing cells and intensity of NFKB2 expression

Due to the enrichment of DEGs in cell cycle signaling pathways from 12-h exposure, this time point was chosen to verify the transcriptome data of *CDKN1A*, *E2F1*, *SERPINE1*, and *NFKB2* at protein level by immunocytochemistry. These genes were selected based on their different functions representing the changes at the molecular biology level. *CDKN1A* is a cell cycle inhibitor, *E2F1* is an important transcription factor for entry in the S-phase, *SERPINE1* plays a role in senescence, and *NFKB2* is a marker for DNA damage [23–26]. As CDKN1A, E2F1 and NFKB2 are assigned to the signaling pathways “immune system” or “cell cycle”, their gene expressions were visualized in Fig. 2. SERPINE1 is an exception and not visualized as it is not assigned to these pathways. It was, however, differentially expressed (6-h exposure to FX-9 FC 5.53, 12-h exposure to FX-9 FC 3.05) and selected as it plays a role in cellular senescence [23]. The time point of 24-h FX-9 exposure was also chosen to monitor the further progression of those proteins.

After 12-h exposure to FX-9, no changes in the amount of CDKN1A-expressing cells, and decreased intensity from moderate to weak-moderate were shown (Table 4). Positively stained E2F1 expressing cells showed slightly stronger intensity but no increase in the fraction of positively stained cells. SERPINE1 expression in the cells had a weak intensity, and the fraction of positively stained cells increased. NFKB2 was expressed by 100% of the cells and showed an increase from moderate-strong to strong intensity after exposure to FX-9. After 24 h of exposure of cells to FX-9, the fraction of CDKN1A-expressing cells increased, while the intensity was constant. In contrast, the fraction of NFKB2-expressing cells showed no variation, although the intensity increased from weak-moderate to moderate-strong. E2F1 and SERPINE1 expressing cells showed no differences in the fraction of positively stained cells and intensity comparing the control cells with the cells exposed to 5 μM.

**Table 4:** ICC-staining comparing control cells with cells after 12-h or 24-h exposure to 5 μM FX-9

		12-h		24-h	
		control	5 μM FX-9	control	5 μM FX-9
CDKN1A (p21)	intensity	m	w-m	m	m
	positively stained cells	+	+	++	+++
E2F1	intensity	w-m	m	w-m	w-m
	positively stained cells	+++	+++	++	++
SERPINE1 (PAI-1)	intensity	w	w	w	w
	positively stained cells	+++	++++	++++	++++
NFKB2	intensity	m-s	s	w-m	m-s
	positively stained cells	++++	++++	++++	++++

Intensity of ICC-staining is categorized into negative (n); weak (w); moderate (m); strong (s). The fraction of positively stained cells are categorized into four groups: + = 0–10% of labeled cells; ++ = 11–40% of labeled cells; +++ = 41–70% of labeled cells; ++++ = 71–100% of labeled cells. Images of ICC-stainings are included into supplementary information (supplementary Figs. 1 and 2).

## Discussion

The aim of the present study was to characterize in vitro the molecular mode of action of FX-9 by gene expression level in a stable in vitro model system (PC-3 cell line) and to verify selected targets at protein level.

At cellular level, pro-apoptotic characteristics of FX-9 could be demonstrated. Gene expression level profiling resulted in DEGs of < 1% of total genes tested comparing the control with the 6-h or 12-h FX-9 exposure. Therefore, the investigation of initial drug-mediated events leading to apoptosis before the expected apoptotic signal overflow was possible. Analyzing the DEGs after 6-h FX-9 exposure showed an inflammatory response, and after the 12-h exposure, cell cycle disturbance. Interestingly, the potent cell cycle inhibitor *CDKN1A* and transcription factor *E2F7* were activated in the 6-h samples, potentially causing the cell cycle-related response in the 12-h samples.

The early response to FX-9 was a stress-induced inflammatory response by activating the nuclear factor-kappa B (NF-κB) signaling pathway. NF-κB includes a family of transcription factors, which have key roles in inflammatory and immune responses [27]. The canonical (classical) NF-κB signaling pathway is activated by stimuli from various immune receptors [27]. Response via the non-canonical (alternative) NF-κB signaling pathway is activated by only a few members of the tumor necrosis factor superfamily receptors and pathogens like viruses and bacteria [28]. MAP3K14 (NIK) has normally a steady level expression in the cytoplasm, and accumulates in response to those activating stimuli. This activates IKKα and phosphorylation of p100 to p52, which forms a complex with RelB [29]. The RelB/p52 complex translocates into the nucleus and activates the transcription of the target genes (Fig. 1). In our study, FX-9 activated the central genes of non-canonical NF-κB signaling such as *MAP3K14* (*NIK*), *RelB*, and *NFKB2*. NF-κB downstream target genes of following classes were activated: cytokines/chemokines and their modulators (*CCL20*, *IL1B*, *IL6*, *IL8* (*CXCL8*)), immunoreceptors (*TNFRSF9*), cell adhesion molecules (*CD44*), regulators of apoptosis (*BIRC2*, *BIRC3*), growth factors, ligands and their modulators (*FSTL3*, *INHBA*, *THBS1*), transcription factors and regulators (*NFKB2*, *RELB*, *NFKBIA*, *TNFAIP3*), and miscellaneous (*CDKN1A*, *GADD45B*) [30]. Of these genes, *TNFAIP3*, encoding protein A20, and *NFKBIA*, encoding protein IκBα, mediate a negative feedback control, resulting in a self-limitating response [31, 32]. In our study, NF-κB activation and inflammatory response were more prominent in the 6-h than in the 12-h samples, which could be caused by the negative feedback mechanism. Activation of NF-κB can mediate anti-apoptotic signals, which leads to survival of the cells [33]. As an example, IL-6 is known for playing a part in chemoresistance and metastasis of aggressive prostate cancer [34]. IL-6 gene expression was increased with an FC of 2.13 after 6 h exposure to FX-9, however, gene expression was not significantly affected after 12-h exposure to FX-9 (FC 1.67). Reduced activation of those signals after 12-h exposure of FX-9 could explain its time dependent pro-apoptotic and anti-proliferative effect observed in a previous study of ours in different cell lines [8].

After 12-h FX-9 exposure, signaling pathways of cell cycle regulation were enriched by DEGs. FX-9 exposure increases the cell cycle inhibitor *CDKN1A*, which encodes protein p21, providing multiple functions, for example, cell cycle arrest in response to drug induced DNA-damage [35]. In our study, similar to other studies, combined upregulation of *CDKN1A* and *E2F7* was shown, and interaction of these genes can repress *E2F1*, a transcription factor for many key proteins, which are essential for G1/S transition [36–38]. Blocking of *E2F1* transcriptional activity inhibits the expression of its target genes [39]. In the present study, *E2F1* was decreased after 12-h exposure to FX-9. The target genes *CDC6* [40], *CDT1* [41], *CCNE2* [42], *MCM3* und *MCM6* [43] were also inhibited (Fig. 2). These genes are essential for transition from the G1-phase to the S-phase, and relevant for forming the pre-replicative complex [44]. Inhibiting these can provide a G1 cell cycle arrest and suppression of DNA replication [45, 46]. These results confirmed the antimitotic character of FX-9 on prostate carcinoma cells. In contrast to the gene expression level profiling, ICC showed a stable E2F1 protein expression. E2F1 protein is inactivated by binding to hypophosphorylated retinoblastoma protein (RB) [47]. Phosphorylation of RB by CDK4/cyclin D in combination with CDK2/cyclin E in the late G1-phase releases E2F1, which subsequently activates genes for G1/S transition [48]. It is possible, that FX-9 inhibited this phosphorylation and that ICC staining detected the inactive E2F1 protein in the RB/E2F1 complex.

FX-9 causes enrichment of DEGs in signaling pathways of cellular senescence in both time points. Morphological studies by Schille et al. showed evidence of a senescent phenotype at the earliest after 12-h FX-9 exposure [8]. Cellular senescence is a persistent cytostasis with a distinct morphological and biochemical phenotype induced by stress stimuli like strong mitotic signals or non-telomeric DNA damage [49]. *SERPINE1* is a marker for senescence and essential for maintenance of this phenotype in fibroblasts [50] and was activated in the present study. Upregulation of the protein PAI-1, encoded by *SERPINE1*, occurred after 12-h FX-9 exposure and showed a stable expression in the 24-h samples compared to the control. This could be due to the fact that in the 24-h samples, 100% of control cells already expressed PAI-1, so no increase in the fraction of positively stained cells could occur. Therapy-induced senescence can be induced by anticancer agents [51], for example, antimitotic agents like microtubule poisons [52]. Senescence is additional evidence for the antimitotic nature of FX-9. Tumor cells could escape from cell cycle arrest and re-enter the cell cycle (mitotic slippage), usually resulting in those senescent cells or cell death in the G1-phase [53]. On a cellular level, this was confirmed by enlarged multinucleated cells [8]. These cells bear tumorigenic potential, which suggests careful consideration should be given to the application of such antimitotic agents [52].

## Conclusions

The molecular acting mechanism of FX-9 was elucidated. Correlation of the observed gene expression changes and the biological response confirmed the antimitotic character of FX-9. Specifically, the enrichment of cell cycle associated DEGs regulating the transition from G1-phase to S-phase supports the thesis of an FX-9 induced G1-phase arrest. Described effects of FX-9 like induction of cell cycle inhibitor *CDKN1A*, activation of NF-κB signaling pathway and senescence can be caused by DNA damage [24, 26, 49]. For the substance family of FX-9, the quinolines, topoisomerase inhibition is a possible mechanism of action, which can cause DNA double strand breaks [54]. The DNA-damaging character and possible topoisomerase inhibition of FX-9 are worth being further evaluated.

## Supplementary Information

**Additional file 1.**

**Additional file 2: Supplementary Fig. 1** Immunocytochemistry staining of control cells and cells after exposure to 5 μM FX-9 for 12 h.

**Additional file 3: Supplementary Fig. 2** Immunocytochemistry staining of control cells and cells after exposure to 5 μM FX-9 for 24 h.

## Data Availability

The raw datasets used and/or analyzed during the current study are available from the corresponding author on reasonable request. The Data of DEGs, which were used for the analysis are available in the additional supporting files.

## Abbreviations

CDKN1A: Cyclin Dependent Kinase Inhibitor 1A
CDKs: Cyclin-dependent kinases
DAVID: Database for Annotation, Visualization and Integrated Discovery
DEGs: Differential expressed genes
FC: Fold change
FDR: False discovery rate
ICC: Immunocytochemistry
PCA: Principal component analysis
TAC: Transcriptome Analysis Console
