# Supplementary figures and images for: Novel chemotherapeutic agent FX-9 activates NF-κB signaling and induces G1 phase arrest by activating CDKN1A in a human prostate cancer cell line
Source: BMC Cancer. 2021 Oct 8;21:1088. doi: 10.1186/s12885-021-08836-y (PMC8501574; doi:10.1186/s12885-021-08836-y)

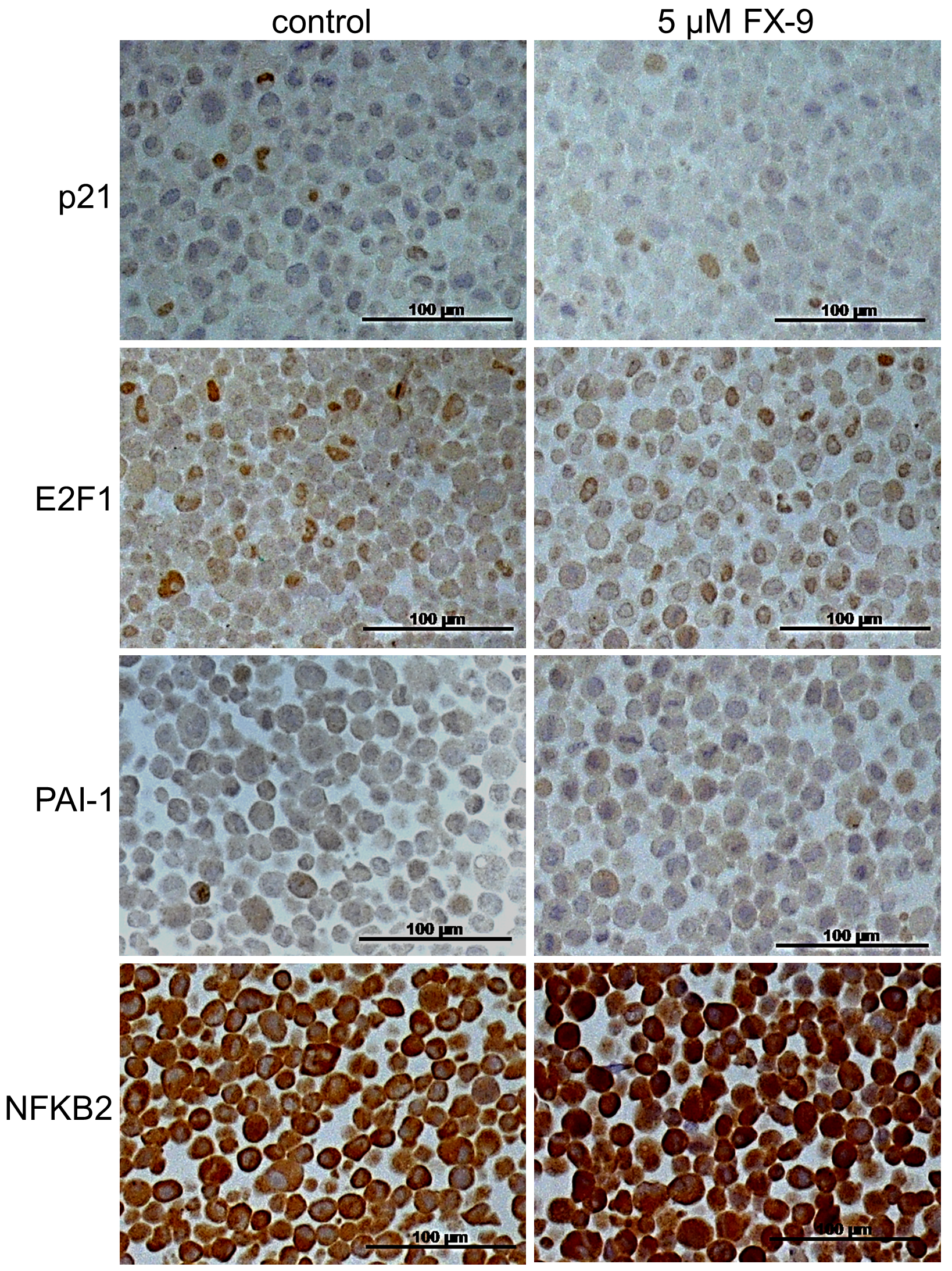

Supplement: Supplementary file 2 — Additional file 2: Supplementary Fig. 1 Immunocytochemistry staining of control cells and cells after exposure to 5 μM FX-9 for 12 h. [file 12885_2021_8836_MOESM2_ESM.tif]

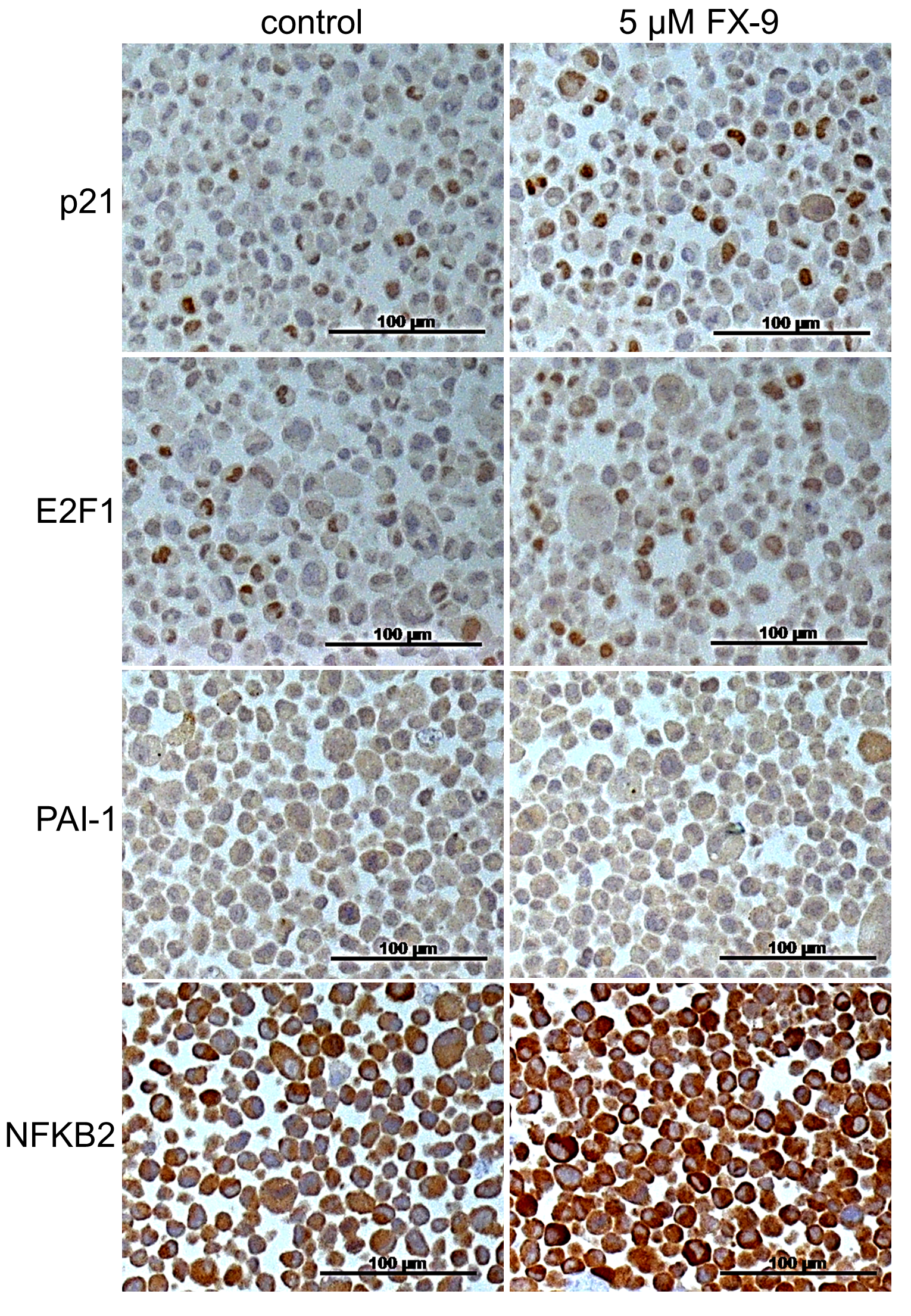

Supplement: Supplementary file 3 — Additional file 3: Supplementary Fig. 2 Immunocytochemistry staining of control cells and cells after exposure to 5 μM FX-9 for 24 h. [file 12885_2021_8836_MOESM3_ESM.tif]
